# On-site workshop investment problem: A novel mathematical approach and solution procedure

**DOI:** 10.1016/j.heliyon.2023.e22678

**Published:** 2023-11-27

**Authors:** Nima Moradi, Vahid Kayvanfar, Roberto Baldacci

**Affiliations:** a Concordia Institute for Information and Systems Engineering, Concordia University, Montreal, Canada; b Division of Engineering Management and Decision Sciences, College of Science and Engineering, Hamad Bin Khalifa University, Qatar Foundation, Doha, Qatar

**Keywords:** On-site workshop, Project scheduling, Multi-mode resource investment problem, Genetic algorithm

## Abstract

In real-world construction sites, On-Site Workshops (OSW) are installed to accelerate construction activities and facilitate the material handling process. These temporary OSWs are cost-effective, leading to decreasing the material handling cost and project makespan, which indicates their important role as a part of a construction project. However, considering the OSW, which has not been addressed in the project scheduling problems, requires the construction site to have a space capacity constraint while considering the workshop size, availability level, and other project-related constraints. In the present work, by considering the OSWs, a real construction project scheduling problem is studied as a Multi-Mode On-Site Workshop Investment Problem with Tardiness (MOSWIPT) while finding the installation/dismantling time of the OSWs. Two new (linear) mathematical programming models are proposed for MOSWIPT. Next, due to the NP-hardness of the problem, an enhanced Genetic Algorithm (GA)-based metaheuristic with efficient problem-specific improvement rules as local search and effective crossover and mutation operators is proposed. Computational experiments show that the proposed method has solved most of the instances of the addressed problem to optimality and outperformed the existing metaheuristics, e.g., Simulated Annealing (SA) and Particle Swarm Optimization (PSO). Finally, conclusions and suggestions for future studies are stated.

## 1 Introduction

Project Scheduling Problem (PSP) has been a popular topic among researchers and decision-makers due to its practical and theoretical importance. PSP seeks to obtain the start time of the activities concerning the constraints of the project and optimize the predetermined objective function. PSP is a very important task since it directly impacts the time, cost, and quality of a project. PSP has two main limitations: (I) the maximum availability level of the resources and (II) the precedence relationship between activities. With these two limitations, the PSP becomes the well-known Resource-Constrained PSP (RCPSP) [1], [2], [3]. The other well-known problem in the PSP is Time-Constrained PSP (TCPSP), which aims to obtain the minimum usage cost of the resources while satisfying the deadline of the project [4].

**Figure 1:**
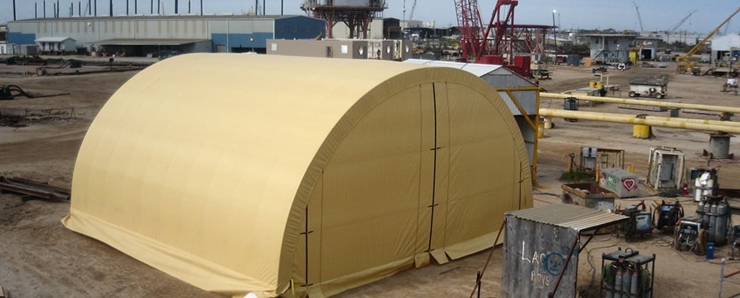
A picture of an OSW at a construction site.

**Figure 2:**
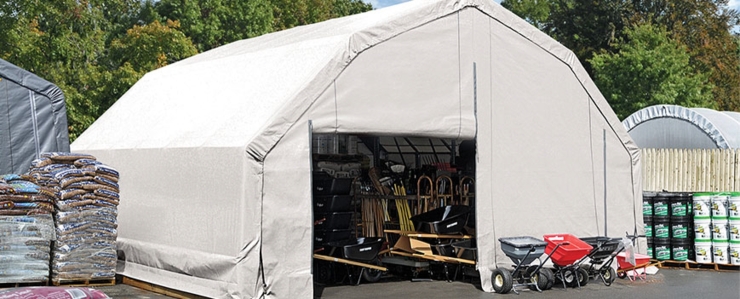
A picture of an OSW used as a material storage place at a construction site.

According to the literature, TCPSP is also known as a Resource Investment Problem (RIP) [5]. RIP determines the level of the resources, minimizing the cost of investment in resources and completing the project in the given time. According to the literature, there are different types of resources in real-world projects, such as renewable (they have limited usage in each period but are renewed next period, e.g., labor, machines, equipment, and space), non-renewable (they are consumed during planning horizon, e.g., money, materials, and energy), doubly-constrained (they are renewable from a period to next period but are non-renewable within each period, e.g., periodic budget), and partially (non)renewable (their presence is connected to specific periods within the planning horizon, and the activities that need these resources will only utilize them when they are executed during these periods [6]). The present work considers a new resource type called an On-Site Workshop (OSW). Each OSW has a specific lifetime —, a period between installing and dismantling times —, and requires a certain place to be located at a construction site. In other words, OSW is a generalized partially (non)renewable resource that poses space capacity constraints in the scheduling process. Therefore, partially (non)renewable resource is a subset of OSWs that does not require a space to occupy. In the construction site, these temporary OSWs are cost-effective, leading to decreasing the material handling cost and project makespan^1^ (as shown in Fig. 1). However, each OSW, which has not been addressed in the construction project scheduling problems, occupies a certain space of the construction site. This requires the construction site to have a (storage) space capacity constraint; for example, as can be seen in Fig. 2, the more OSWs (with a certain size) are established, the more space the construction site occupies.

Image source: https://www.shelterlogic.com/knowledge/site-workshops-storage-shelter-industry.

Image source: https://www.shelterlogic.com/knowledge/site-workshops-storage-shelter-industry.

Thus, the occupied space by OSWs is an important factor that must be considered since, in the real world, OSWs are built next to each other, and the total space available to install these workshops is limited at the construction site [7], [8]. Also, the OSW's lifetime directly impacts the project scheduling/planning phase because some project activities rely on a specific OSW to be started. Considering the OSW's limited availability, the sum of the occupied space of OSW by activities can not be greater than the OSW's available amount. Moreover, each OSW is a temporary workshop used to carry out some construction project activities. Hence, each OSW has a particular function: storage, cutting, welding, painting workshops, etc. Installing and dismantling such a temporary workshop, which has a limited available level, poses an issue for the activities' start/finish time as well as the makespan of the project. In addition, not only does each OSW have a limited availability, but the construction site also has a certain space capacity, resulting in applying a limited number of OSWs simultaneously.

This paper presents a new problem in the context of PSP: Multi-Mode On-Site Workshop Investment Problem with Tardiness (MOSWIPT), which aims to find the optimal lifetime and the occupied level of OSWs, activities start time, and execution mode of each activity so that usage of OSWs and tardiness penalty costs are minimized while satisfying the space capacity of the construction site, resource-constrained and precedence relationships constraints. This paper is organized as follows: The second section reviews the literature. The third section states the problem and presents the mathematical models for the problem. The fourth section describes the proposed solution procedure. The fifth section represents the computational experiments to evaluate the performance of the proposed models and solution techniques. Finally, the sixth section states the conclusion and suggestions for future studies.

## 2 Literature review

### 2.1 Resource investment problems

Three characteristics identify the PSP: (I) resources, which are classified as renewable, non-renewable, partially renewable, or fixed, stochastic, uncertain, time-dependent, etc., (II) activities, such as single-mode, multi-mode, (non)preemptive, deterministic, stochastic, uncertain, fuzzy, time-dependent, resource-dependent, etc., (III) objective function, such as, minimizing the project makespan, resource leveling, minimizing the resource investment, maximizing the Net Present Value (NPV), etc. Due to these various characteristics, [9] suggested a classification scheme to denote the PSP problems with unanimous notations and symbols. The present paper complies with the notations introduced by [9] to classify the MOSWIPT (see section 3).

If a penalty is assigned to the tardiness of the project completion time, then RIP becomes RIP with Tardiness Penalty (RIPT) [10]. If the tardiness penalty is considered a constraint, not an objective function, then RIPT is called the Resource Availability Cost Problem (RACP) [11]. RIPT/RACP has been studied by both exact and heuristics solvers, including Branch-and-Bound [12], Lagrangian Relaxation and Column Generation (CG) [5], Constraint Programming (CP) [13], Genetic Algorithm (GA) [10], [14], Particle Swarm Optimization (PSO) [15], Artificial Immune System (AIS) [16].

Due to the theoretical and practical importance of RIPT/RACP, several variants have been introduced in the literature, such as Multi-Mode RIP (MRIP) [17], [18], RIP with Time Windows [19], RIP with Quantity Discount Problem [20], Multi-Mode Preemptive RIPT [21], Fuzzy-Stochastic RIP [22]. Moreover, [23] extended the MRIP to multi-agent settings, proposing decentralized negotiation mechanisms to facilitate the allocation of global resources. [24] presented a novel interval programming and chance-constrained optimization-based hybrid solution approach for a fully uncertain, multi-objective, and MRIP scheduling problem. [21] studied the preemptive MRIP minimizing the total (non)renewable resource costs and earliness-tardiness costs by a given project deadline and activity due dates. Also, a Mixed Integer Programming (MIP) formulation and GA are proposed for the problem. [25] studied the MRIP with Tardiness Penalty (MRIPT) by proposing a Large Neighborhood Search (LNS) where destroy operators were applied to a feasible solution to obtain subproblems. Then, these subproblems were solved with MIP-based recreate operators to obtain an improved solution. Our addressed problem, MOSWIPT, extends MRIPT by considering partially (non)renewable resources and space capacity constraints.

### 2.2 Project scheduling with storage space capacity

Limited storage space has emerged as a significant bottleneck in projects carried out in urban areas. Some recent works have considered space capacity constraints in the construction project scheduling. For example, [26] addressed the integrated RCPSP and Material Procurement Scheduling (MPS). While previous literature had primarily focused on solving the simultaneous solution of RCPSP-MPS with one warehouse (storage) and unlimited capacity, they introduced a novel approach that considered multiple warehouses with limited capacity over the entire planning horizon. They employed a metaheuristic method, namely population-based Simulated Annealing (SA), to find acceptable solutions efficiently within a short time frame. Also, [27] studied the Project Scheduling and Material Ordering Problem (PSMOP) with storage space constraints. Their proposed model minimized the makespan of the project, material inventory, ordering, and indirect costs by finding the activity schedule and the material ordering time and quantity. Also, they designed an efficient, non-dominated sorting GA (NSGA-II) to solve the problem for large-sized instances. Recently, [28] tackled the integrated RCPSP and PSMOP with Limited Storage Space (RCPSMOP-LSS) regarding the activity scheduling and material ordering when faced with storage constraints. They introduced a Two-Layer Heuristic Algorithm (DLHA) to solve the model. The addressed problem in the present paper, MOSWIPT, differs from RCPSP-MPS or RCPSMOP-LSS by employing partially (non)renewable resources and parallelized activities and OSWs.

### 2.3 Project scheduling under partially (non)renewable resources

Also, the partially (non)renewable resource concept was first introduced by [6], which encompasses traditional renewable and nonrenewable resource constraints. The primary objective was to minimize the makespan of the PSP under partially (non)renewable resources. They used an enumeration method for exact solutions and provided bounds considering future resource consumption for faster convergence. [29] discussed the historical focus of project scheduling research on developing solution methods and generalizing models, neglecting the generation of problem instances until recently, e.g., partially (non)renewable resources. Furthermore, they highlighted the limitations of the classical RCPSP when dealing with labor time regulations in manpower scheduling scenarios. Furthermore, [30] introduced a novel heuristic algorithm rooted in Scatter Search (SS) for addressing PSP under partially (non)renewable resources. The efficacy of the SS algorithm was evaluated using established test instances.

Moreover, [31] presented a set of preprocessing techniques and multiple heuristic algorithms tailored to address PSP under partially renewable resources. They introduced heuristic algorithms based on Greedy Randomized Adaptive Search Procedure (GRASP) and path relinking, which were tested using existing test instances and demonstrated excellent performance. Also, [32] proposed an Integer Programming (IP) model and a Constraint Programming (CP) model for PSP under partially renewable resources and resource consumption during setup operations. They introduced a heuristic method, termed the “mask calculation algorithm,” which restricted selectable modes to enhance the efficiency of the search process.

In recent work, [33] addressed RCPSP, incorporating partially renewable resources and general temporal constraints. They introduced the concept of partially renewable resources within the context of projects with broader temporal constraints, enhancing its applicability to real-world project scenarios. They presented a branch-and-bound procedure and developed new consistency tests, lower bounds, and dominance rules. Similarly, [34] introduced a novel branch-and-bound algorithm for solving RCPSP with partially renewable resources and general temporal constraints, employing a stepwise decomposition approach to analyze potential resource consumption by project activities. This method effectively limited the depth of the enumeration tree, achieving polynomial complexity through a binary search mechanism. The addressed problem in the present paper, MOSWIPT, extends the multi-mode RACP with recruitment and release dates for resources (MRACP-RR) [21] and PSP under partially renewable resources by considering the space capacity of the construction site and introducing the decision variables on parallelized activities and OSWs.

**Figure 3:**
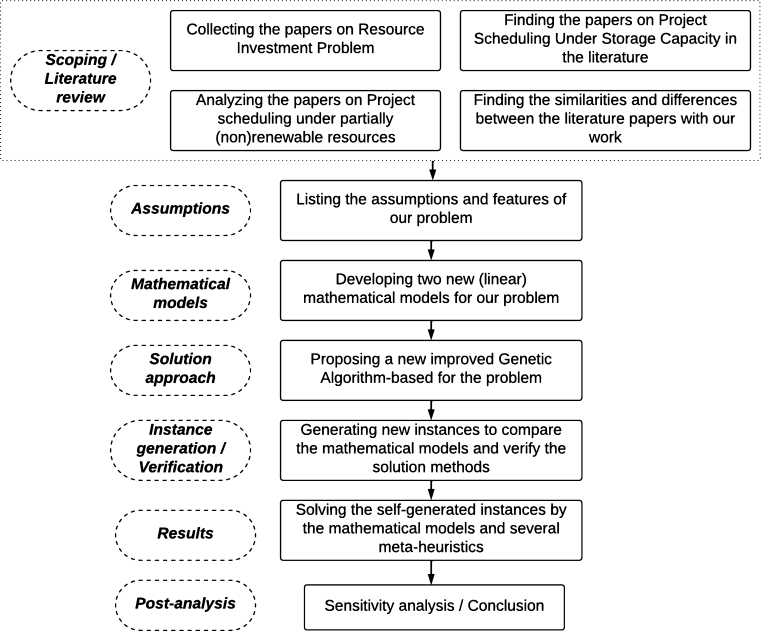
Research methodology steps of the present paper.

The main contribution of the present work is to extend the well-known MRIPT (or MRACP), PSMOP, and PSP with partially (non) renewable resources by taking the lifetime and the spatial constraints of the OSW into account while satisfying the space capacity of the construction site. Hence the contributions of the present work can be listed as follows: (I) A new problem in the context of project scheduling is presented: multi-mode OSW investment problem with tardiness, or MOSWIPT, finding the optimal lifetime and occupied level of OSWs, activities start time and execution mode of each activity; (II) Two new linear mathematical models are proposed for MOSWIPT; one with time-indexed decision variables and the other one with variables for parallelized activities and OSWs, (III) A GA-based metaheuristic is proposed for MOSWIPT, which is enhanced by problem-specific improvement rules and efficient crossover/mutation operators and compared with SA and PSO algorithms over the new instances generated for MOSWIPT according to the dataset at PSPLIB (http://www.om-db.wi.tum.de/psplib/). For ease of understanding the various problems in the literature, the list of abbreviations is provided in Table 1. Moreover, the research methodology steps in this work are presented in Fig. 3. In the methodology of this paper, the first step involved conducting a literature review to gain a deep understanding of the problem domain and identify existing research gaps. Subsequently, two linear mathematical models were developed to model the problem, providing a theoretical foundation for the proposed solutions. A GA-based metaheuristic was proposed to enhance the effectiveness and efficiency of these models. To empirically evaluate the proposed models and the metaheuristic, various problem instances were generated, and experiments were conducted to measure their performance. Furthermore, sensitivity analysis was carried out to assess the models' performance and identify critical parameters influencing their outcomes.

**Table 1:** List of abbreviations.

Abbreviation	Phrase
PSP	Project Scheduling Problem
RCPSP	Resource-Constrained PSP
TCPSP	Time-Constrained PSP
RIP	Resource Investment Problem
OSW	On-Site Workshop
MOSWIPT	Multi-Mode On-Site Workshop Investment Problem with Tardines
RIPT	RIP with Tardiness Penalty
RACP	Resource Availability Cost Problem
MRIP	Multi-Mode RIP
MRIPT	MRIP with Tardiness Penalty
MPS	Material Procurement Scheduling
PSMOP	Project Scheduling and Material Ordering Problem
RCPSMOP-LSS	RCPSP and PSMOP with Limited Storage Space
MRACP-RR	Multi-Mode RACP with Recruitment and Release Dates for Resources

## 3 Problem statement and formulation

MOSWIPT contains activities of the Finish-to-Start (FS) type, where some activities occupy a certain space of the OSWs to be started. The total sum of these occupied spaces must not be greater than the maximum space capacity of related OSWs; the total sum of the occupied spaces by the OSWs must not be greater than the space capacity of the construction site. The assumptions of the addressed problem, MOSWIPT, are given as follows: (I) each activity occupies a certain space of an OSW; (II) Some OSWs may not be able to be constructed at the same time due to the space limitations of the construction site; (III) The occupied space by each OSWs is constant and varied from one to the other; (IV) OSW-related cost includes the installation (usage) cost per unit; (V) OSWs and project activities occupy a continuous space; (VI) an individual OSW cannot be split into separated parts; (VII) There is a space capacity for the construction site at where OSWs are located; (VIII) Each OSW has unique application, so they are not interchangeable; (IX) Duration time of each activity is fixed; (X) Project activities have more than one execution modes (multi-mode) and are non-preemptive; (XI) Precedence relationships are the FS type; (XII) OSWs may be installed right after the first activity, which needs this OSW, becomes active, and they may be dismantled right after the last activity, which needs this OSW, becomes inactive; (XIII) Delay in project completion has a penalty.

**Figure 4:**
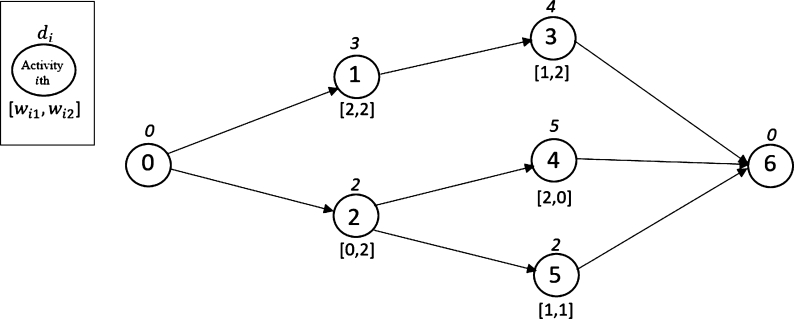
The AON project network for the numerical example.

**Figure 5:**
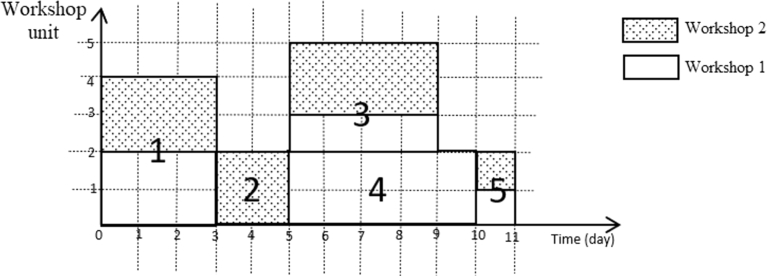
A feasible schedule for the numerical example when the space capacity of the construction is unlimited.

**Figure 6:**
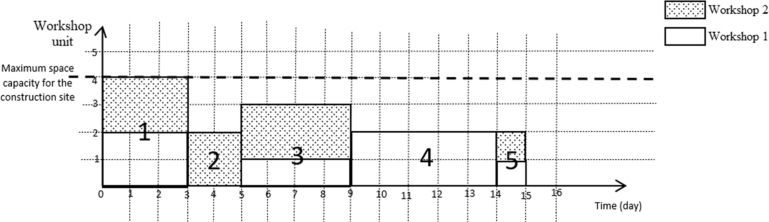
A feasible schedule for the numerical example when the space capacity of the construction is limited to four units.

To explain the MOSWIPT in more detail, a numerical example is given in this section. Assume a project with five activities (with a single execution mode) and two OSWs. The Activity-on-Node (AON) network for this project with the parameters is given in Fig. 4. In Fig. 4, $w_{i1}$ is the amount of space that activity *i* occupies in the first OSW; also, $w_{i2}$ is the amount of space that the activity *i* occupies in the second OSW, and $d_{i}$ is the duration of activity *i*. Now, assume that the construction site has unlimited space capacity and the space capacity of each OSW is three units. A feasible schedule for this project is given in Fig. 5. This feasible schedule indicates that the project is finished on the 11th day, and the space capacity of each OSW is not violated. On the other hand, if we assume that the construction site has a limited space capacity of 4 units, then the schedule given in Fig. 5 becomes an infeasible solution since, in the period between the fifth and ninth days, the sum of the amount of space occupied by both OSWs is greater than four units. The feasible schedule for the case of assuming the space capacity for the construction site is given in Fig. 6. In the new schedule, the project is finished on the 15th day (4 days more than the previous schedule) while satisfying the space capacity of each OSW and construction site. This example clearly shows that by assuming a limited space capacity for the construction site, project scheduling is affected dramatically; for example, the makespan of the project in 11 units (days) increases to 15, as shown in Figure 5, Figure 6, respectively.

MOSWIPT can be represented by a directed graph (AON network) G=(V,E), in which there are N+2 activities (nodes), and arcs are the FS precedence relationship between activities. The initial and terminal activities are dummies, so there are *N* main activities. Also, each activity j∈J=V with the execution mode of $i\in m_{j}$ ($m_{j}$ is the set of possible execution modes for activity j∈J), has a fixed duration $d_{ji}$. Furthermore, each activity j∈J with the execution mode of $i\in m_{j}$ requires a fixed space of each OSW k∈K equals $r_{jik}$ (*K* is the set of available OSWs). In addition, the precedent activities of activity j∈J are given by the set $P_{j}$. Also, the construction site has a fixed space capacity of *Q*, which cannot be violated. Moreover, the usage cost of the OSW per unit and the tardiness penalty are presented by $C_{k}$ and $C_{d}$, respectively. Also, the time units are defined by the set of T={0,1,...,H} (*H* is the planning horizon), and the deadline of the project is $T_{max}$. According to the notations introduced by [9], MOSWIPT is symbolized as $1,v,va|cpm,\delta_{n},mu|rac,T_{max}$.

Two (linear) mathematical programming models are proposed for MOSWIPT, MP1-MOSWIPT, and MP2-MOSWIPT. The main difference between the two models is the definition of their decision variables. In MP1-MOSWIPT, the optimal values for the activities' start time, installing and dismantling times of each OSW, and execution mode of each activity are found by binary decision variables. In MP2-MOSWIPT, the optimal activities start time and installing/dismantling times of each OSW are defined as non-negative continuous decision variables besides finding the parallel activities and OSW.

### 3.1 The first proposed mathematical model for MOSWIPT

The parameters and decision variables of MP1-MOSWIPT are in Table 2. This model finds the optimal values of the activities' start time, the occupied level of OSWs, the installing and dismantling times of each OSW, and the execution mode of each activity while satisfying the precedence relationship, OSW's size, and capacity of construction site constraints. The first linear formulation, MP1-MOSWIPT, which is developed based on the proposed formulations by [17], [35], [25], [18] and modified for the addressed problem, is presented as follows:

(1) $$Min\;\sum_{k\in K}C_{k}R_{k}+C_{d}D$$

subject to,

(2) $$\sum_{t\in T}tx_{n+1,t}-T_{max}⩽D$$

(3) $$\sum_{u\in m_{j}}z_{ju}d_{ju}+\sum_{t\in T}tx_{jt}⩽\sum_{t\in T}tx_{it}\;\;\;\forall i\in J,\forall j\in P_{i}$$

(4) $$\sum_{i\in J}\sum_{j\in m_{i}}r_{ijk}y_{ijkt}^{\prime }⩽R_{k}\;\;\;\forall k\in K,\forall t\in T$$

(5) $$y_{ijkt}^{\prime }+1⩽z_{ij}+y_{kt}\;\;\;\forall i\in J,\forall j\in m_{i},\forall k\in K,\forall t\in T$$

(6) $$y_{ijkt}^{\prime }⩽\beta (z_{ij}+y_{kt})\;\;\;\forall i\in J,\forall j\in m_{i},\forall k\in K,\forall t\in T$$

(7) $$\sum_{k\in K}R_{kt}^{\prime }⩽Q\;\;\;\forall t\in T$$

(8) $$0⩽R_{kt}^{\prime }⩽R_{k}\;\;\;\forall k\in K,\forall t\in T$$

(9) $$R_{kt}^{\prime }⩽My_{kt}\;\;\;\forall k\in K,\forall t\in T$$

(10) $$R_{kt}^{\prime }⩽R_{k}-M(1-y_{kt})\;\;\;\forall k\in K,\forall t\in T$$

(11) $$tx_{it}+\sum_{j\in m_{i}}d_{ij}z_{ij}-1⩽\sum_{t^{\prime }\in T}y_{kt^{\prime }}\;\;\;\forall j\in J,\forall k\in K,\forall t\in T$$

(12) $$\sum_{j\in m_{i}}z_{ij}=1\;\;\;\forall i\in J$$

(13) $$\sum_{t\in T}x_{it}=1\;\;\;\forall i\in J$$

(14) $$x_{00}=1$$

(15) $$x_{it},z_{ij},y_{kt},y_{ijkt}^{\prime }\in \{0,1\},\;\;\;\forall i\in J,\forall j\in m_{i},\forall k\in K,\forall t\in T$$

**Table 2:** Parameters and decision variables of the first mathematical model, MP1-MOSWIPT.

Sets:	
*J*	Set of activities including initial and terminal (dummy) activities; *j* ∈ *J* = {0,1,2,...,*N*,*N* + 1}
*m*_*j*_	Set of possible execution modes for activity *j* ∈ *J*
*K*	Set of available OSWs (resources) *k* ∈ *K*
*P*_*j*_	Set of the precedent activities of activity *j* ∈ *J*
*T*	set of time units; *t* ∈ *T* = {0,1,...,*H*}

**Parameters:**	
*N*	Number of project (non-dummy) activities
*d*_*ji*_	Duration of activity *j* executed my mode *i* ∈ *m*_*j*_
*r*_*jik*_	The amount of space that activity *j* ∈ *J* with the execution mode of *i* ∈ *m*_*j*_ needs to occupy in OSW *k* ∈ *K*
*Q*	Space capacity of the construction site
*C*_*k*_	Usage cost of the OSW *k* ∈ *K* per unit
*C*_*d*_	Penalty cost for each unit of tardiness
*H*	Planning horizon
*T*_*max*_	Project deadline

**Decision variables:**	
*x*_*it*_	A binary variable equals 1 if activity *i* ∈ *J* starts at time *t* ∈ *T*; 0 otherwise.
*z*_*ij*_	A binary variable equals 1 if activity *i* ∈ *J* is executed by the mode *j* ∈ *m*_*i*_; 0 otherwise.
*y*_*kt*_	A binary variable equals 1 if the OSW *k* ∈ *K* is active at time *t* ∈ *T*; 0 otherwise.
$y_{ijkt}^{\prime }$	A binary variable equals 1 if the activity *i* ∈ *J* with mode *j* ∈ *m*_*i*_ occupies the OSW *k* ∈ *K* at time *t* ∈ *T*; 0 otherwise.
*R*_*k*_	Availability level of the OSW *k* ∈ *K* during the project.
$R_{kt}^{\prime }$	Availability level of the OSW *k* ∈ *K* at time *t* ∈ *T*.

In MP1-MOSWIPT, the objective function (1) minimizes the usage costs of OSWs plus the penalty for tardiness, in which *D* is a non-negative continuous decision variable; the objective function (1) and constraints (2) linearize the non-linear form of $D=Max\{0,\sum_{t\in T}tx_{n+1,t}-T_{max}\}$. Constraints (3) ensure the precedence relationships between the activities. Constraints (4)-(6) ensure that at each time unit, the occupied space of the OSW must not be greater than the availability level of that OSW. Also, these constraints (4)-(6) linearize the non-linear constraints of $\sum_{i\in J}\sum_{j\in m_{i}}z_{ij}r_{ijk}y_{kt}⩽R_{k},\forall k\in K,\forall t\in T$, in which *β* is an arbitrary constant parameter such that 0<β<1. Constraints (7)-(10) satisfy the space capacity of the construction site, and they linearize the non-linear constraints of $\sum_{k\in K}R_{k}y_{kt}⩽Q,\forall t\in T$. Note that in constraints (9)-(10), *M* is a big constant parameter and chosen such that $0⩽R_{k}⩽M,\forall k\in K$. Constraints (11) enforce that the OSW must be installed when an activity needs it and remain in use until the activity is not finished. Constraints (12) ensure that only one execution mode must be chosen for each activity. By constraints (13), each activity starts one time. Constraint (14) forces the initial activity to start at time 0. The domain of the decision variables is given by constraints (15).

### 3.2 The second proposed mathematical model for MOSWIPT

In the second proposed mathematical model, MP2-MOSWIPT, a novel model in the literature, the possibility of parallelization of activities and OSWs is considered rather than the lifetime of OSWs and activities. Decision variables of MP2-MOSWIPT are given in Table 3. MP2-MOSWIPT finds the optimal scheduling for activities and OSWs and considers the parallelized activities and OSWs as binary variables. In other words, MP2-MOSWIPT focuses on parallel activities and OSWs to minimize the usage cost of the OSW and the tardiness penalty. Also, MP2-MOSWIPT needs to determine the required OSW for each activity; thus, the set $H_{k}$ is defined as the activities that require the OSW k∈K. The second proposed mathematical model, MP2-MOSWIPT, is presented as follows:

**Table 3:** Decision variables of the second mathematical model, MP2-MOSWIPT.

Decision variables:	
*A*_*iu*_	A binary variable equals 1 if activity *i* ∈ *J* is parallelized with activity *u* ∈ *J*; 0 otherwise. (*A*_*ii*_ = 1 means activity *i* is being executed during the project)
$A_{iju}^{\prime }$	A binary variable equals 1 if activity *i* ∈ *J* with mode *j* ∈ *m*_*i*_ is parallelized with the activity *u* ∈ *J*; 0 otherwise.
$W_{kk^{\prime }}$	A binary variable equals 1 if the OSW *k* ∈ *K* is parallelized with the OSW *k*′ ∈ *K*; 0 otherwise. (*W*_*kk*_ = 1 means the OSW *k* is being activated during the project)
$W_{kk^{\prime }}^{\prime }$	Availability level of the OSW *k* ∈ *K* while it is parallelized with the OSW *k*′ ∈ *K*
*S*_*i*_	Start time of activity *i* ∈ *J*
*SW*_*k*_	Installing time of the OSW *k* ∈ *K*
*FW*_*k*_	Dismantling time of the OSW *k* ∈ *K*
*ϕ*,*ϕ*′,*ω*,*ω*′	Continuous decision variables defined for simplification of the model.

The objective function (1) subject to (2), (12), and:

(16) $$S_{i}⩽S_{j}+\sum_{u\in m_{j}}z_{ju}d_{ju}\;\;\;\forall i\in J,\forall j\in P_{i}$$

(17) $$\sum_{i\in J}\sum_{j\in m_{i}}r_{ijk}A_{iju}^{\prime }⩽R_{k}\;\;\;\forall k\in K,\forall u\in J$$

(18) $$A_{iju}^{\prime }+1⩽z_{ij}+A_{iu}\;\;\;\forall i,u\in J,\forall j\in m_{i}$$

(19) $$A_{iju}^{\prime }⩽\lambda (z_{ij}+A_{iu})\;\;\;\forall i,u\in J,\forall j\in m_{i}$$

(20) $$\sum_{k\in K}W_{kk^{\prime }}^{\prime }⩽Q\;\;\;\forall k^{\prime }\in K$$

(21) $$0⩽W_{kk^{\prime }}^{\prime }⩽R_{k}\;\;\;\forall k^{\prime }\in K$$

(22) $$W_{kk^{\prime }}^{\prime }⩽MW_{kk^{\prime }}\;\;\;\forall k,k^{\prime }\in K$$

(23) $$W_{kk^{\prime }}^{\prime }⩽R_{k}-M(1-W_{kk^{\prime }})\;\;\;\forall k,k^{\prime }\in K$$

(24) $$0⩽SW_{k}⩽S_{i}\;\;\;\forall i\in H_{k},\forall k\in K$$

(25) $$FW_{k}⩽S_{i}+\sum_{j\in m_{i}}z_{ij}d_{ij}\;\;\;\forall i\in H_{k},\forall k\in K$$

(26) $$A_{iu}⩽\phi \;\;\;\forall i,u\in J$$

(27) $$\phi ⩽S_{u}-S_{i}+1\;\;\;\forall i,u\in J$$

(28) $$A_{iu}⩽\omega \;\;\;\forall i,u\in J$$

(29) $$\omega ⩽S_{i}+\sum_{j\in m_{i}}z_{ij}d_{ij}-S_{u}+1\;\;\;\forall i,u\in J$$

(30) $$W_{kk^{\prime }}⩽\phi^{\prime }\;\;\;\forall k,k^{\prime }\in K$$

(31) $$\phi^{\prime }⩽FW_{k^{\prime }}-SW_{k}+1\;\;\;\forall k,k^{\prime }\in K$$

(32) $$W_{kk^{\prime }}⩽\omega^{\prime }\;\;\;\forall k,k^{\prime }\in K$$

(33) $$\omega^{\prime }⩽FW_{k}-FW_{k^{\prime }}+1\;\;\;\forall k,k^{\prime }\in K$$

(34) $$A_{iu},A_{iju}^{\prime },W_{kk^{\prime }},z_{ij}\in \{0,1\},\phi ,\phi^{\prime },\omega ,\omega^{\prime }⩽0\;\;\;\forall i,u\in J,\forall k,k^{\prime }\in K,\forall j\in m_{i}$$

In MP2-MOSWIPT, constraints (16) ensure the precedence relationships between the activities. Constraints (17)-(19) ensure that the occupied space of an OSW must not be greater than the availability level of that OSW. Also, the constraints (17)-(19) linearize the non-linear constraints of $\sum_{i\in J}\sum_{j\in m_{i}}z_{ij}r_{ijk}A_{iu}⩽R_{k},\forall k\in K,\forall u\in J$. Note that in constraints (19), *λ* is an arbitrary constant parameter, where 0<λ<1. Constraints (20)-(23) satisfy the space capacity of the construction site, and they also linearize the non-linear constraints of $\sum_{k\in K}R_{k}W_{kk^{\prime }}⩽Q,\forall k^{\prime }\in K$. Note that in constraints (22) and (23), *M* is a big constant parameter chosen such that $0⩽R_{k}⩽M,\forall k\in K$. Constraints (24) ensure that its required OSW must be installed before starting an activity. Constraints (25) ensure that the OSW cannot be dismantled unless the activities needed for it are finished. Constraints (26)-(29) ensure that a pair of activities can be parallelized if the start time of one of them is between the start and finish time of the other one. Constraints (30)-(33) ensure that a pair of OSWs can be parallelized if the finish time of one is between the start and finish time of the other. The domain of the decision variables is given by constraints (34).

The number of constraints of MP1-MOSWIPT is $O(\overset{¯}{K}T(n+m^{\prime }))$, where n, T, $\overset{¯}{K}$ and $m^{\prime }$ are the number of activities, the number of periods, the number of precedent activities of all activities, the number of available OSWs, and the number of execution modes of all activities, respectively. Also, the number of decision variables of MP1-MOSWIPT is $O(T(\overset{¯}{K}m^{\prime }+n))$. Also, for MP2-MOSWIPT, the number of constraints is $O(n(\overset{¯}{K}+m^{\prime }+n)+{\overset{¯}{K}}^{2})$, and the number of decision variables of MP2-MOSWIPT is $O(n(m^{\prime }+n)+{\overset{¯}{K}}^{2})$. Therefore, the proposed linear models are polynomial in the number of constraints and decision variables.

## 4 An enhanced Genetic Algorithm for MOSWIPT

If the project activities become single-mode, and the capacity space for installing the OSWs turns to infinity, then MOSWIPT reduces to the RIPT. It has been proven that RIPT is NP-hard [12], so the MOSWIPT is also NP-hard. Due to the proven efficiency of GA-based solvers for PSPs, this paper proposes an enhanced GA-based solution technique to solve the MOSWIPT in an acceptable time when large instances cannot be solved optimally.

**Figure 7:**
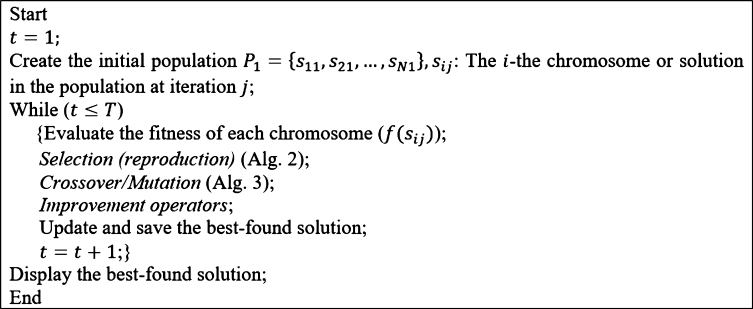
Alg. 1: Pseudo-code of the proposed GA for MOSWIPT.

**Figure 8:**
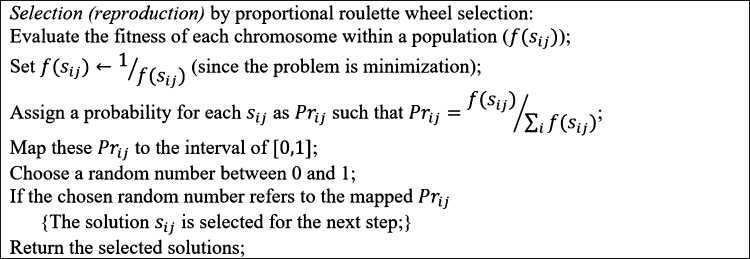
Alg. 2: The *selection* step in the proposed GA.

**Figure 9:**
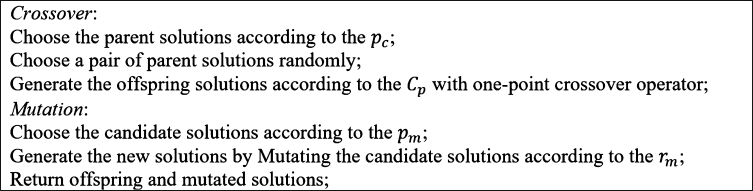
Alg. 3: The *crossover/mutation* step in the proposed GA.

GA was first proposed by John Holland and explained completely in [36]. GA goes through a stochastic search to improve the solutions to the problem and seeks to improve the generated solutions while keeping the best-found solution consisting of chromosomes (genotype), a population, a fitness function, and a local search. Chromosomes represent the problem's solution throughout the solution space and encode the solutions to be understandable by GA. The population is a set of solutions generated at each iteration of GA. The fitness function indicates a better solution in terms of the objective function. Also, local search is an important element of GA defining the operators to generate the neighbor solutions; for example, crossover and mutation operators are well-known tools to search the neighborhood of a solution. Generally, the crossover operator explores the solution space by finding far solutions (diversification), and the mutation operator exploits the solution space by finding the close (local) solutions (intensification). Making a trade-off between diversification and intensification is critical to increasing the efficiency of the proposed GA, which becomes possible by tuning the GA parameters. The parameters of GA include the size of the population (*N*), the probability of crossover ($p_{c}$), the probability of mutation ($p_{m}$), the number of generations (*T*), the mutation rate ($r_{m}$), and the crossover point ($C_{p}$) of one-point crossover. The pseudo-code of the proposed GA for MOSWIPT, the *selection*, and *crossover/mutation* steps are presented in Figure 7, Figure 8, Figure 9, respectively. The main contribution of the proposed GA is in employing problem-specific improvement operators, which makes the GA capable of finding high-quality solutions while avoiding low-quality solutions.

### 4.1 Solution representation

To encode a solution of MOSWIPT by the chromosome, this paper proposes the following representation, where *n*, and $\overset{¯}{K}$ are the number of activities and OSWs, respectively:

**Chromosome:** $\left\{S_{1},...,S_{n},SW_{1},...,SW_{\overset{¯}{K}},FW_{1},...,FW_{\overset{¯}{K}},M_{1},...,M_{n},R_{1},...,R_{\overset{¯}{K}}\right\}$

**Figure 10:**
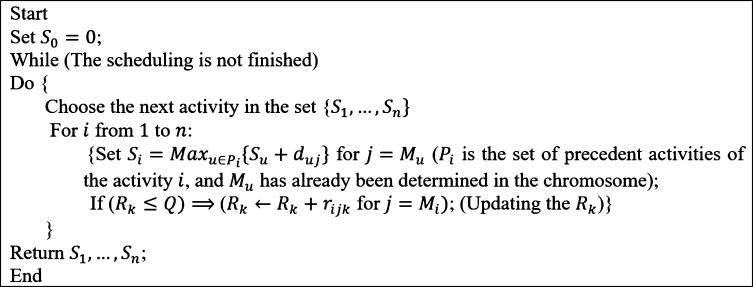
Alg. 4: The algorithm of finding the feasible schedule for the project activities.

So, the chromosome in the proposed GA is a ($2n+3\overset{¯}{K}$)-dimensional vector, in which $\left\{S_{1},...,S_{n}\right\}$, $\left\{SW_{1},...,SW_{\overset{¯}{K}}\right\}$, $\left\{FW_{1},...,FW_{\overset{¯}{K}}\right\}$, $\left\{M_{1},...,M_{n}\right\}$, and $\left\{R_{1},...,R_{\overset{¯}{K}}\right\}$ are the start time of the activities, the install time of the OSW, the dismantle time of the OSW, the execution mode of the activities, and the availability level of the OSW, respectively. To generate the initial population, each chromosome must be a feasible solution that satisfies the constraints of the precedence relationships, the space capacity, and the maximum available level of the OSWs. At first, each $R_{k}$ (k∈K) is found by assigning an integer random number greater than $max_{i\in J,j\in m_{i}}\{r_{ijk}\}$. Next, each $M_{i}$ (i∈J) is found by assigning an integer random number from the set $m_{i}$, the activity's execution mode *i*. Then, the start time of the activities is found by Alg. 4, as given in Fig. 10. Finally, each $SW_{k}$ and $FW_{k}$ are found according to the activities which need the OSW k∈K to be started, or mathematically by Eq. (35):

(35) $$SW_{k}=min_{i:\exists j\in m_{i},r_{ijk}\neq 0}\{S_{i}\}\;\text{and}\;FW_{k}=max_{i:\exists j\in m_{i},r_{ijk}\neq 0}\{S_{i}+d_{ij}\}$$

### 4.2 Calculation of the fitness function

The fitness function or objective function value of a chromosome (solution) consists of two parts: (I) The usage cost of the OSWs and (II) The tardiness penalty cost. The first part can be calculated simply by multiplying each $R_{k}$ and $C_{k}$ for all k∈K. For the second part, the completion time of the project or $S_{n+1}$ is found by $S_{n+1}=S_{n}+d_{nj}$ where $j=M_{n}$; as a result, the objective (fitness) function is found by $\sum_{k\in K}C_{k}R_{k}+C_{d}\times max\{0,S_{n+1}-T_{max}\}$.

### 4.3 Crossover operator

The input of the crossover operator is two chromosomes, known as parent solutions, and its output is two chromosomes, known as offspring solutions. Assume that there are two parent solutions as follows:

1st parent solution: $\left\{S_{1}^{1},...,S_{n}^{1},SW_{1}^{1},...,SW_{\overset{¯}{K}}^{1},FW_{1}^{1},...,FW_{\overset{¯}{K}}^{1},M_{1}^{1},...,M_{n}^{1},R_{1}^{1},...,R_{\overset{¯}{K}}^{1}\right\}$ 2nd parent solution: $\left\{S_{1}^{2},...,S_{n}^{2},SW_{1}^{2},...,SW_{\overset{¯}{K}}^{2},FW_{1}^{2},...,FW_{\overset{¯}{K}}^{2},M_{1}^{2},...,M_{n}^{2},R_{1}^{2},...,R_{\overset{¯}{K}}^{2}\right\}$

Now a point is chosen according to the parameter of $C_{p}$, then the crossover operates on that point and generates the offspring solutions as follows (For example, assume the crossover point is between the elements (genes) $n+\overset{¯}{K}$ and $n+\overset{¯}{K}+1$ with the sign of △):

1st parent solution: $\left\{S_{1}^{1},...,S_{n}^{1},SW_{1}^{1},...,SW_{\overset{¯}{K}}^{1}△FW_{1}^{1},...,FW_{\overset{¯}{K}}^{1},M_{1}^{1},...,M_{n}^{1},R_{1}^{1},...,R_{\overset{¯}{K}}^{1}\right\}$ 2nd parent solution: $\left\{S_{1}^{2},...,S_{n}^{2},SW_{1}^{2},...,SW_{\overset{¯}{K}}^{2}△FW_{1}^{2},...,FW_{\overset{¯}{K}}^{2},M_{1}^{2},...,M_{n}^{2},R_{1}^{2},...,R_{\overset{¯}{K}}^{2}\right\}$

Then, the offspring solutions are generated as follows:

1st offspring: $\left\{S_{1}^{1},...,S_{n}^{1},SW_{1}^{1},...,SW_{\overset{¯}{K}}^{1}△FW_{1}^{2},...,FW_{\overset{¯}{K}}^{2},M_{1}^{2},...,M_{n}^{2},R_{1}^{2},...,R_{\overset{¯}{K}}^{2}\right\}$ 2nd offspring: $\left\{S_{1}^{2},...,S_{n}^{2},SW_{1}^{2},...,SW_{\overset{¯}{K}}^{2}△FW_{1}^{1},...,FW_{\overset{¯}{K}}^{1},M_{1}^{1},...,M_{n}^{1},R_{1}^{1},...,R_{\overset{¯}{K}}^{1}\right\}$

Note that it is possible that the offspring solutions would be infeasible after operating the crossover. So, the crossover operator is designed to repair the infeasible solutions and convert them into feasible ones. If the crossover changes the start time of the activities, then Alg. 4 repairs the schedule. If the crossover changes the schedule of the OSWs (both installing and dismantling times), then the infeasible schedule for the OSW is repaired by Eq. (35). Suppose the crossover changes the execution mode of each activity and the availability level of the OSWs. In that case, the scheduling of the activities and the OSWs are updated and repaired according to the new execution modes by Alg. 4.

### 4.4 Mutation operator

The input of the mutation operator is one chromosome, and its output is a mutated chromosome. The parameter of $r_{m}$ determines the number of genes (bits) mutated in each chromosome. For example, assume that the following chromosome is a solution to be mutated:

Before mutation: $\left\{S_{1}^{1},...,S_{n}^{1},SW_{1}^{1},...,SW_{\overset{¯}{K}}^{1},FW_{1}^{1},...,FW_{\overset{¯}{K}}^{1},M_{1}^{1},...,M_{n}^{1},R_{1}^{1},...,R_{\overset{¯}{K}}^{1}\right\}$

Now assume that the elements *n* and $n+\overset{¯}{K}$ are mutated, then the output will be as follows (new genes are $S_{n}^{\prime \;1}$ and $SW_{\overset{¯}{K}}^{\prime \;1}$):

After mutation: $\left\{S_{1}^{1},...,S_{n}^{\prime \;1},SW_{1}^{1},...,SW_{\overset{¯}{K}}^{\prime \;1},FW_{1}^{1},...,FW_{\overset{¯}{K}}^{1},M_{1}^{1},...,M_{n}^{1},R_{1}^{1},...,R_{\overset{¯}{K}}^{1}\right\}$

Like the crossover operator, it is possible that after mutation, the output solution would be infeasible. So, the mutation operator is designed to avoid infeasible solutions. Suppose the mutation changes the scheduling of an activity and the availability level of the OSWs. In that case, the start time of the activity is changed to a value satisfying the precedence relationship and the space capacity constraints according to Alg. 4. If the mutation changes the scheduling of the OSW, then the installing or dismantling time of the OSW is changed to a value satisfying the feasible schedule of the activities according to Eq. (35). Finally, suppose the mutation changes the execution modes. In that case, the execution modes are chosen among the available execution modes for that activity, satisfying the feasible schedule of the activities and the space capacity according to Alg. 4. After the crossover/mutation step, the resultant population goes through the improvement phase for possible improvements. The final population is saved and updated in the next iterations until returned as the best-found optimal solution in the last step of GA.

### 4.5 Problems-specific *improvement* operators

**Figure 11:**
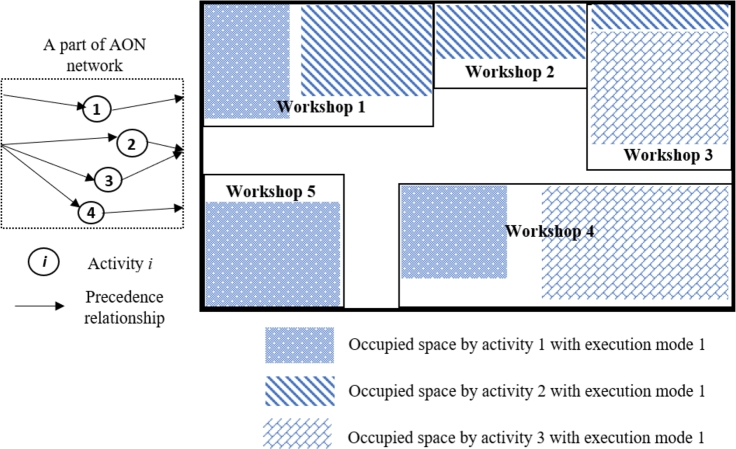
A construction site, occupied with five workshops and the AON network (a numerical example), when activity one is executed with mode 1.

**Figure 12:**
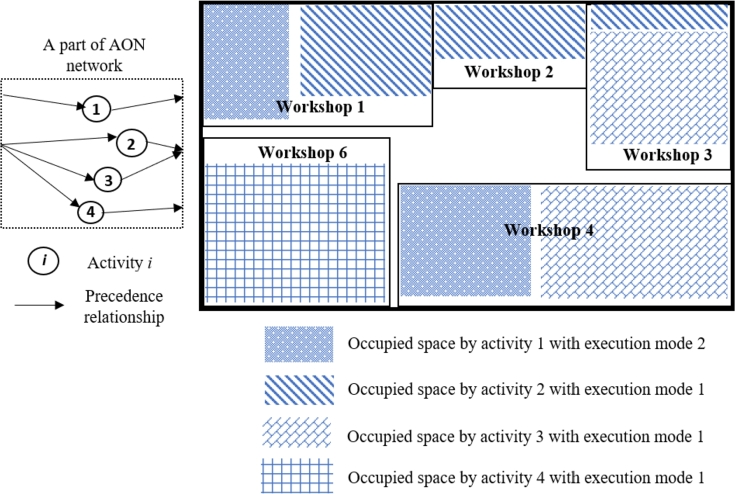
The construction site, occupied with five workshops and the AON network, when the execution mode of activity one is switched to mode two by *Io*_1_.

In this section, three problem-specific *improvement* operators are designed to enhance performance within the proposed GA. These three MOSWIPT-specific improvement operators are defined based on the well-known improvement rules in the literature (as given in Table 4). In other words, the improvement rules, whose efficiency is proven in the literature, are modified and adapted to the newly addressed problem, MOSWIPT. The first improvement operator, $Io_{1}$, changes the execution mode of each activity to decrease the makespan by parallelizing the activities or to decrease the occupied space of an OSW to make it possible for other activities to be activated while not violating the space capacity of the construction site. For example, consider a construction site where five OSWs are installed, and OSWs 1, 4, and 5 are occupied by activity 1; also, activities 2 and 3 occupy workshops {1,2,3} and {3,4}, respectively (Fig. 11). The precedence relationships for this example are given on the left-hand side of Fig. 11. Now, the first improvement operator ($Io_{1}$) switches the execution mode of activity one from 1 to 2. Execution mode 2 requires activity 1 to occupy more space in workshop four while there is no need to occupy any space in workshop 5 (see Fig. 12); therefore, activity four is made parallel with activities 1, 2, and 3, according to the precedence relationships given in Fig. 11, since activity four only needs workshop six and total space capacity of the construction site is not violated as well. By parallelizing the four activities in this example, the makespan of the project is decreased while satisfying the constraints.

**Table 4:** Problem-specific *improvement* operators employed within the proposed GA.

*Improvement* operator	Symbol	Definition
Execution mode switch	*Io*_1_	The execution mode of the activities is switched while not violating the space capacity of the construction site and improving the objective function.
Activities rescheduling	*Io*_2_	The start time of the activities are rescheduled according to their float time while improving the objective function.
Regularization of the availability level of OSW	*Io*_3_	The availability level of each OSW is changed (regularized) while not becoming lower than the required space of the OSW by the activities.

**Figure 13:**
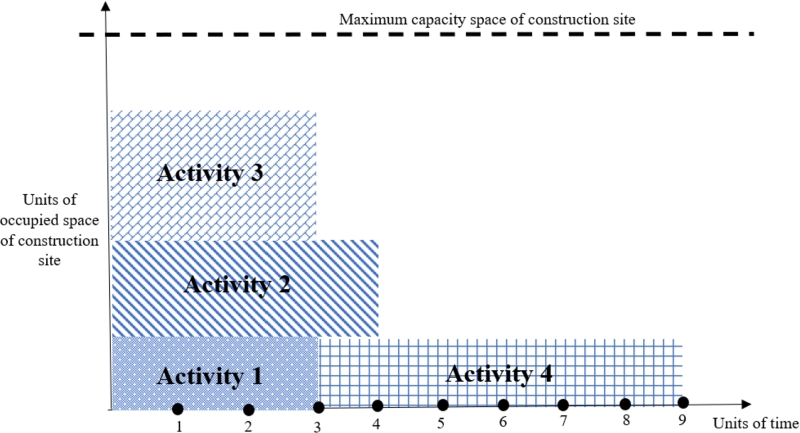
Activities schedule of the numerical example when activity 1 starts at time zero.

**Figure 14:**
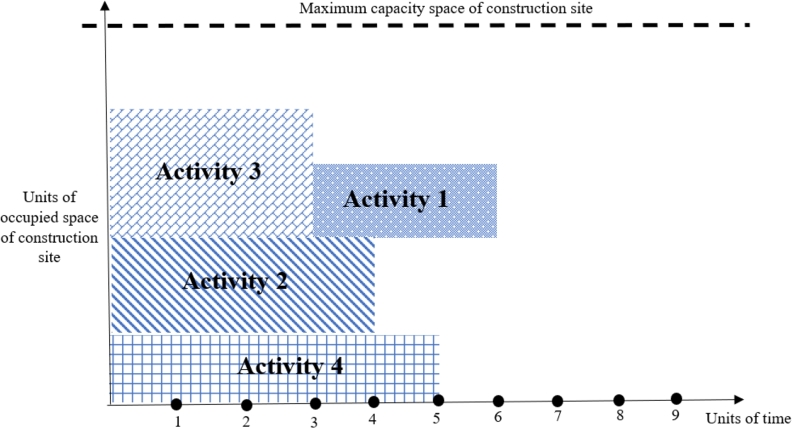
Activities schedule of the numerical example when activity one is rescheduled to start at time three by *Io*_2_.

The second improvement operator, $Io_{2}$, reschedules the activities according to their float times to decrease the makespan while not violating the project constraints, including precedence relationships and construction site space capacity. Let's consider the example given in Fig. 11, where the duration of activities 1, 2, 3, and 4 are 3, 4, 3, and 6, respectively. Considering the limited space capacity of the construction site, activity four cannot start unless one of activities 1, 2, or 3 finishes. The schedule for this project is given in Fig. 13. Since activity 1 is not a successor of activities 2 or 3, the second improvement operator ($Io_{2}$) reschedules its start time from 0 to 3 units of time while satisfying the space capacity constraint (Fig. 14). The new schedule decreases activities' finish time from 9 to 6 units of time, decreasing the objective function value.

**Figure 15:**
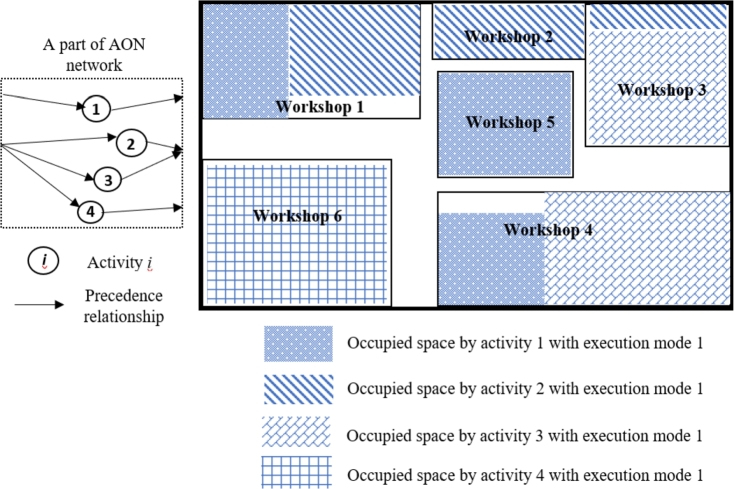
The construction site, occupied with six workshops, when the size of each OSW is regularized by *Io*_3_.

Finally, the third improvement operator ($Io_{3}$) regularizes the size (availability level) of each OSW to include more workshops on the construction site if possible. For example, consider the construction site given in Fig. 11. The third improvement operator ($Io_{3}$) reduces the size of each OSW while keeping the required space for activities to occupy each OSW. After regularizing the sizes of each workshop, available space for installing the OSW 6 is created to be occupied by activity 4 (Fig. 15). Thus, more workshops are installed, more activities are parallelized, and the objective function is decreased.

## 5 Computational results

This section presents several comprehensive experiments, including the comparison of mathematical models, sensitivity analysis of GA parameters, performance evaluation of the different versions of GA, and comparison of the proposed GA with other metaheuristics. All proposed linear mathematical models were solved by the Python-Gurobi interface. Also, GA was coded in C++ programming language using a 1.60 GHz Intel Core i5 processor with 16 GB RAM.

### 5.1 Instance generation for MOSWIPT

In the MP1-MOSWIPT and MP2-MOSWIPT, several new parameters are presented in the literature for the first time. So, in this section, these new parameters are determined to generate the various instances of MOSWIPT. The parameters of MOSWIPT are close to the well-known MMRCPSP [37]. To generate the instances for MOSWIPT, three sets of parameters, including the usage cost of each OSW ($C_{k}$), penalty for the tardiness ($C_{d}$), and the amount of required space of the OSW k∈K by the activity *i* with the execution mode $j\in m_{i}$ ($r_{ijk}$) were added to the MMRCPSP library.^2^ The instances of *c15, c21, j10, j12, j14, j16, j18, j20, j30, m2*, and *r3* are chosen from this library. First, the behavior of the problem concerning the parameters was investigated, so 16 instances of *j10* were chosen. Next, the parameters $r_{ijk}$ were assigned a number from 0 to 13 ascendingly, while the remaining parameters were unchanged. Like $r_{ijk}$, parameters $C_{k}$ and $C_{d}$ were assigned a number among 10, 20, 30, 50, and 100 ascendingly, while the remaining parameters were unchanged. The results of these instances of *j10* showed that higher values for $r_{ijk}$ led to a situation in which the OSW and the activities were not able to be parallelized with other OSWs or activities, so the tardiness of the project increased. Moreover, the results showed that higher $r_{ijk}$ increased the $R_{k}$ if the $C_{d}$ was greater than $C_{k}$, in which the optimal solution tended to have a non-parallelized OSW and activities. This means that the $C_{d}$ had more effect on the objective function than $C_{k}$ when $r_{ijk}$ had higher values. Accordingly, three parameters of $r_{ijk}$, $C_{k}$, and $C_{d}$ of MOSWIPT were generated according to the values of rand(0,13), rand(10,30) and 20, respectively (rand(a,b) was a function generating a random number between a and b).

### 5.2 Comparison of MP1-MOSWIPT and MP2-MOSWIPT

At first, the various instances of MOSWIPT were solved by an exact commercial solver, Gurobi, coded by Python language programming. The computational results of MP1-MOSWIPT and MP2-MOSWIPT are given in Table 5; some instances of each dataset were not solved optimally within the maximum execution time of 3600 seconds ($T^{\prime }$ is the execution time in seconds). The difference between the number of executed instances and the number of solved instances is that when the execution time of an instance got higher than 3600 seconds, that instance was considered an unsolved problem. Table 5 shows that MP2-MOSWIPT is faster than MP1-MOSWIPT in most instances, even in large-size instances (*j30*). Also, MP2-MOSWIPT could solve more instances to optimality in comparison with MP1-MOSWIPT; the execution time of MP2-MOSWIPT was raised in some datasets (*j12, j18, j20, j30, r3*).

**Table 5:** The results by the exact solver, Gurobi, over the various-sized instances of MOSWIPT.

Dataset	Models	Minimum *T*′	Maximum *T*′	Average of *T*′	Number of executed instances	Number of instances solved optimally
*c15*	MP1-MOSWIPT	17	2750	1105	34	34
	MP2-MOSWIPT	15	2530	1040	34	34
*c21*	MP1-MOSWIPT	3.4	3600	680	84	77
	MP2-MOSWIPT	1.3	3600	620	84	78
*j10*	MP1-MOSWIPT	0.5	480	21	536	536
	MP2-MOSWIPT	0.4	470	20	536	536
*j12*	MP1-MOSWIPT	0.9	3600	35	101	100
	MP2-MOSWIPT	0.9	3600	39	101	101
*j14*	MP1-MOSWIPT	2.5	2505	610	117	117
	MP2-MOSWIPT	1.3	2110	545	117	117
*j16*	MP1-MOSWIPT	4.6	3600	330	45	41
	MP2-MOSWIPT	5.6	3600	335	45	41
*j18*	MP1-MOSWIPT	5	3600	2120	93	68
	MP2-MOSWIPT	8	3600	2450	93	70
*j20*	MP1-MOSWIPT	25	3600	470	64	37
	MP2-MOSWIPT	33	3600	662	64	39
*j30*	MP1-MOSWIPT	98	3600	2080	10	2
	MP2-MOSWIPT	132	3600	2509	10	3
*m2*	MP1-MOSWIPT	2.4	3600	660	89	75
	MP2-MOSWIPT	2.2	3600	630	89	75
*r3*	MP1-MOSWIPT	3	3600	550	113	95
	MP2-MOSWIPT	9.8	3600	755	113	96

### 5.3 Sensitivity analysis of GA parameters

**Figure 16:**
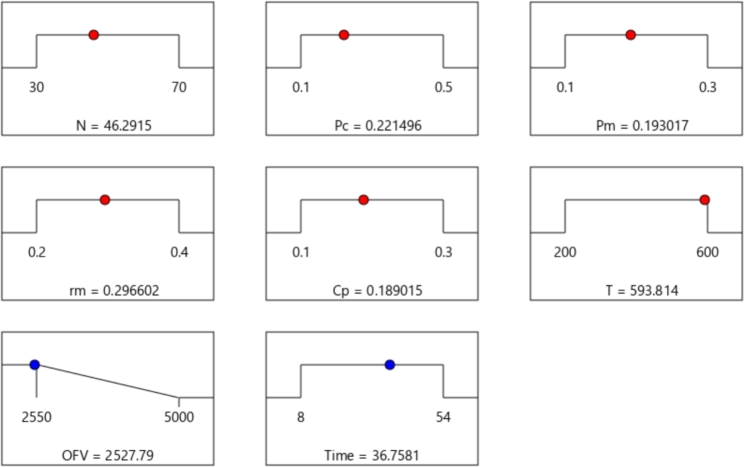
Best values of the parameters of GA for MOSWIPT found by DOE.

**Figure 17:**
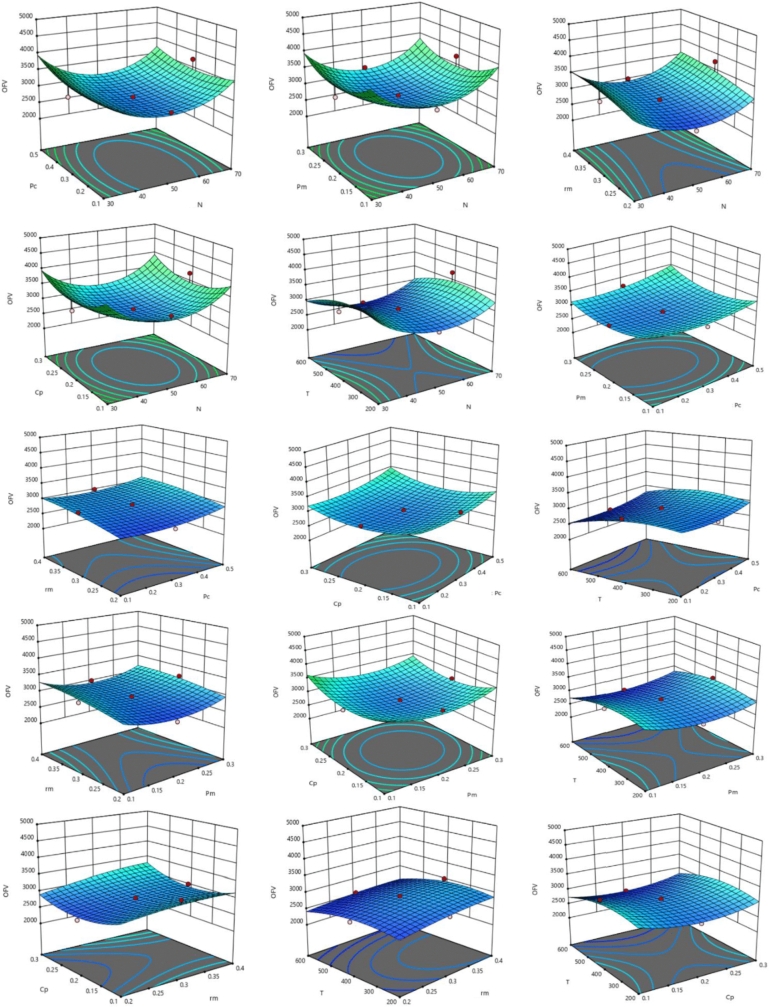
The behavior of the objective function value (*OFV*) with respect to the pair of the GA parameters.

According to the design of experiments (DOE) by response surface methodology (RSM), the optimal parameters of the proposed GA were obtained on the instances of dataset *c15* (each instance of dataset *c15* was executed five times). The range and optimal values for the parameters of GA were presented in Fig. 16, which were returned by the Design-Expert. 11 software. The range of *N* was between 30 and 70, while DOE found the optimal value of 46.2915 for *N* (since the population size, *N*, is an integer value, we chose N=50). Also, by Fig. 16, the initial range of $P_{c}$, $P_{m}$, $r_{m}$, $C_{p}$, and *T* were [0.1,0.5], [0.1,0.3], [0.2,0.4], [0.1,0.3], and [200,600], respectively; in addition, the optimal values of $P_{c}$, $P_{m}$, $r_{m}$, $C_{p}$, and *T* were obtained as 0.3, 0.2, 0.3, 0.2, and 600, respectively. In addition, the behavior of the objective function value (*OFV*) for the various pairs of parameters of GA is presented in Fig. 17. Also, Fig. 17 shows that if *N* equals 50, then by setting $p_{m}=0.2$, the *OFV* becomes lower; or when N=50 and $p_{c}=0.3$, *OFV* is decreased.

### 5.4 Performance evaluation of the proposed GA

In this section, the results of the proposed GA are presented. For this experiment, the parameters of GA are considered as follows: $N=50,p_{c}=0.3,p_{m}=0.2,r_{m}=0.3,C_{p}=0.2,T=600$. First, the instances of MMRCPSP at the PSPLIB, including *c15, c21, j10, j12, j14, j16, j18, j20, j30, m2*, and *R3*, were extracted, and then the new parameters were added to the instances of each dataset. To evaluate the performance of the GA in MOSWIPT instances, three improvement rules (operators) were added to GA to enhance its efficiency (Table 4).

Finally, the results of the comparative study of different versions of GA with SA [38] and PSO [39] over the MOSWIPT instances are given in Table 6. In this experiment, the execution time was limited to 60 seconds. Also, in this experiment, the parameters of GAs were tuned to $N=50,p_{c}=0.3,p_{m}=0.2,r_{m}=0.2,C_{p}=0.2$, and the parameters of SA were $T_{max}=1000,T_{min}=0.01,\alpha =0.98,N=20$, (*α*: Cooling rate, *N*: The number of iteration at each temperature) and the parameters of PSO were adjusted to $n_{s}=20,c_{1}=c_{2}=2,M=100$, ($n_{s}$: Swarm size, $c_{1},c_{2}$: two learning factors, *M*: Maximum total number of iterations). Moreover, three criteria are used to compare the performance of the GA, SA, and PSO, namely, the number of instances solved to optimality ($N^{⁎}$), an average of relative difference percentage between returned solutions and the optimal solutions ($A^{⁎}$), or mathematically $A^{⁎}=\frac{f_{i}-f_{o}}{f_{o}}\times 100$, where $f_{i}$ and $f_{o}$ are the objective functions returned in the *i*th iteration, and the optimal objective function, respectively; and finally, the last criterion is the average execution time of the algorithm ($T^{⁎}$). In Table 6, five versions of GA are provided as follows: (I) $GA_{1}$: GA without any improvement rule, (II) $GA_{2}$: GA will all improvement rules except $Io_{3}$, (III) $GA_{3}$: GA will all improvement rules except $Io_{2}$, (IV) $GA_{4}$: GA will all improvement rules except $Io_{1}$, (V) $GA_{5}$: GA with all improvement rules.

**Table 6:** The comparative study of the proposed GA with the existing metaheuristic over the various-sized instances of MOSWIPT.

Dataset	Number of instances	Criteria	*GA*_1_	*GA*_2_	*GA*_3_	*GA*_4_	*GA*_5_	SA	PSO
*c15*	34	*N*^⁎^	17	20	20	22	25	15	17
		*A*^⁎^	3.52	3.07	3.14	2.99	2.24	4.04	3.66
		*T*^⁎^	47.09	47.33	49.19	52.01	55.18	54.01	49.33
*c21*	84	*N*^⁎^	20	24	22	24	30	18	20
		*A*^⁎^	5.88	5.12	5.24	5.10	3.96	6.33	6.10
		*T*^⁎^	56.22	56.33	57.10	57.09	59.75	49.11	54.88
*j10*	536	*N*^⁎^	490	510	500	510	515	440	480
		*R*^⁎^	0.97	0.77	0.71	0.76	0.53	3.88	1.98
		*T*^⁎^	46.22	50.12	50.01	54.33	57.19	47.11	49.01
*j12*	101	*N*^⁎^	82	88	85	88	90	75	80
		*A*^⁎^	1.88	1.39	1.44	1.37	0.91	2.87	2.25
		*T*^⁎^	46.22	50.87	50.91	49.01	55.01	41.08	46.88
*j14*	117	*N*^⁎^	92	95	95	99	99	81	87
		*A*^⁎^	2.02	1.87	1.91	1.66	1.53	2.90	2.51
		*T*^⁎^	50.09	54.78	54.88	58.03	58.99	45.98	52.99
*j16*	45	*N*^⁎^	21	23	22	23	25	16	19
		*A*^⁎^	4.33	4.10	4.02	4.31	2.77	5.01	4.76
		*T*^⁎^	53.11	55.98	55.87	56.88	58.90	50.09	51.12
*j18*	93	*N*^⁎^	35	38	36	38	40	29	30
		*A*^⁎^	9.66	9.12	9.18	8.87	8.32	11.61	11.01
		*T*^⁎^	51.12	54.88	54.12	55.61	58.91	45.11	49.10
*j20*	64	*N*^⁎^	25	29	29	33	35	22	25
		*A*^⁎^	8.99	8.10	8.16	7.61	7.22	9.79	9.10
		*T*^⁎^	50.11	53.29	53.90	55.01	58.96	43.11	49.05
*j30*	10	*N*^⁎^	1	1	1	2	2	1	1
		*A*^⁎^	10.21	9.05	9.51	6.61	6.05	10.09	9.83
		*T*^⁎^	54.09	58.09	57.77	58.98	59.16	49.02	52.49
*m2*	89	*N*^⁎^	45	49	48	52	55	40	42
		*A*^⁎^	5.67	5.01	5.14	4.87	4.51	6.33	6.19
		*T*^⁎^	50.22	54.98	53.09	56.77	59.09	42.01	49.88
*r3*	113	*N*^⁎^	50	53	52	56	60	45	48
		*A*^⁎^	7.51	7.11	7.42	6.78	6.20	8.21	7.88
		*T*^⁎^	53.44	55.91	54.01	57.09	59.56	49.01	52.01

**Figure 18:**
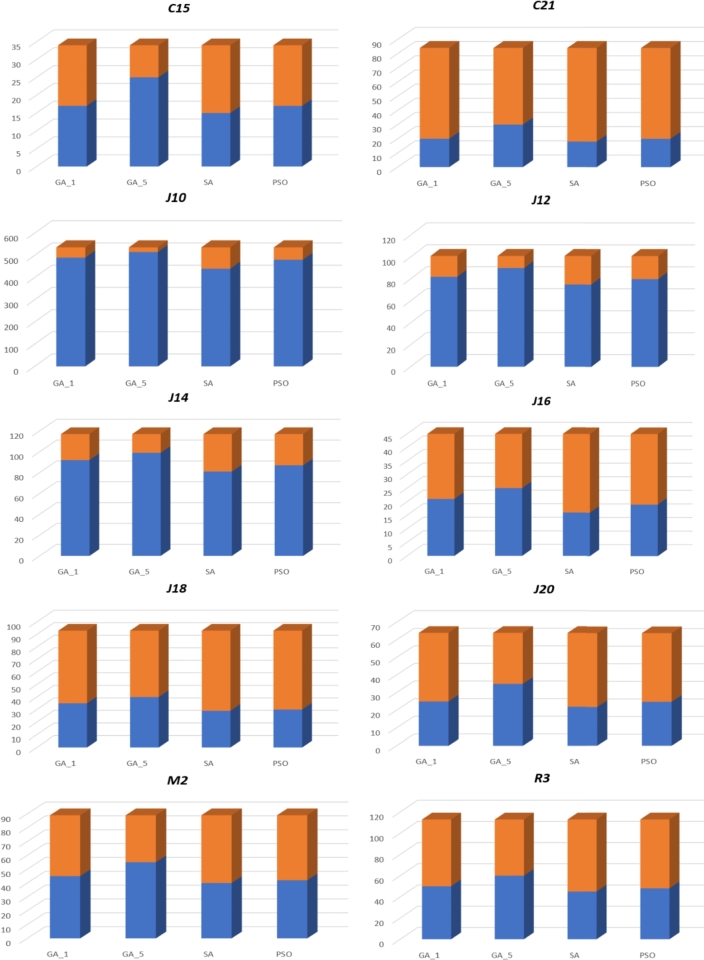
The comparison of the enhanced GA with the existing solvers in terms of total instances solved to optimality (Vertical axis: The number of instances).

In Table 6, $GA_{5}$, the GA with all improvement rules, outperforms all remaining algorithms in terms of both the number of the optimal solution found ($N^{⁎}$) and the average of closeness to the optimal solution ($A^{⁎}$) in small- and large-size instances. However, its execution time is higher than most of the algorithms, which is explainable in that since $GA_{5}$ applies all improvement rules, it takes more time to improve a solution at each iteration compared to the other GA versions. Also, since $A^{⁎}$ is small for most algorithms, it is expected that by increasing the upper bound of the execution time, GA could decrease the gap between the near-optimal solution and the optimal (global) solution. Moreover, Table 6 proves the effectiveness of the improvement rules in applying them to the GA for solving the MOSWIPT; meanwhile, the various versions of GA have better performance than the other two metaheuristics, SA and PSO, in most instances which shows the efficiency of the employed local search operators like *crossover/mutation* operators. Furthermore, Fig. 18 compares the proposed GA ($GA_{5}$) with the existing metaheuristic in terms of the number of instances solved to the optimality in each dataset; the blue parts show the instances that have been solved to the optimality. Also, Fig. 18 shows that the enhanced GA with improvement rules has obtained more optimal solutions compared to the other solvers (it has a larger blue area in each dataset in Fig. 18), which confirms its efficiency in solving the MOSWIPT instances.

### 5.5 Discussion on results

In the end, some remarks can be concluded as follows: (I) GA with all improvement rules outperforms the other versions of GA, SA and PSO in terms of the number of instances solved to optimality (best-known solutions) and the closeness to the optimal solution (best-found solutions) although its execution time is higher than most of the algorithms, (II) All enhanced versions of GA outperform the GA without any improvement rule, (III) GA with all improvement rules except the second rule has worse performance than the other versions GA, which shows that the second rule is significant in enhancing the performance of the GA in terms of the improvement rules, (IV) The mathematical model with the decision variables of parallelized activities and OSW outperforms the first mathematical model in terms of both the number of instances solved to optimality and execution time, (V) Designed *improvement* rules and *crossover/mutation* operators are efficient in obtaining the near-optimal solutions for different instances.

## 6 Conclusion and suggestions for future studies

The present paper studies a new problem in the context of PSP, called MOSWIPT, which aims to find the optimal lifetime, a period between installing and dismantling time, of the OSW at the construction site, the availability level of the OSW, activities start time and execution mode of each activity. Also, the objective function of MOSWIPT is to minimize the usage cost of OSWs and the tardiness penalty while satisfying the construction site's project-related constraints and space capacity. Moreover, two new linear mathematical models are proposed for MOSWIPT: one with time-indexed decision variables and the other with variables for parallelized activities and OSWs. Due to the NP-hardness of the problem, GA-based metaheuristics enhanced by efficient improvement rules and effective crossover and mutation operators are proposed to obtain the solutions with high quality for large-sized instances. Finally, computational experiments show that GA with all improvement rules outperforms the other versions of GA, SA, and PSO regarding the number of instances solved to optimality and the closeness to the optimal solution, although its execution time is higher.

For future studies, there are promising areas. First, valid inequalities and lifting methods can strengthen the proposed mathematical models. Second, finding the optimal location for each OSW can be considered to extend the problem and make it close to real-world projects. Third, considering the problem's parameters as stochastic, uncertain, or fuzzy numbers can make the problem more real. Fourth, integrating MOSWIPT with the facility layout planning problem [40], [41] can be an interesting topic. Finally, state-of-the-art metaheuristics such as Grey Wolf Optimizer (GWO) [42], Enhanced Intelligent Water Drops (EIWD) and Cuckoo Search (CS) [43], and Artificial Immune Systems (AIS) [44] can be implemented in the addressed problem.

## Funding

This research received no specific grant from any funding agency in the public, commercial, or not-for-profit sectors.

## CRediT authorship contribution statement

**Nima Moradi:** Writing – review & editing, Writing – original draft, Visualization, Software, Methodology, Formal analysis. **Vahid Kayvanfar:** Writing – review & editing, Writing – original draft, Validation, Supervision, Project administration, Methodology, Investigation, Data curation, Conceptualization. **Roberto Baldacci:** Writing – review & editing, Validation, Supervision, Data curation, Conceptualization.

## Declaration of Competing Interest

The authors declare that they have no known competing financial interests or personal relationships that could have appeared to influence the work reported in this paper.

## Data Availability

Some or all data, models, or codes that support the findings of this study are available from the corresponding author upon reasonable request.

## Footnotes

1 https://www.shelterlogic.com/knowledge/site-workshops-storage-shelter-industry.

2 http://www.om-db.wi.tum.de/psplib/getdata_mm.html.
